# Treatment of Gleet and Sexual Neurasthenia by Means of Metallig Bougies

**Published:** 1891-04

**Authors:** Charles Szadek

**Affiliations:** Kieff, Russia


### TREATMENT OF GLEET AND SEXUAL NEURASTHENIA BY MEANS OF METALLIC BOUGIES.

BY CHARLES SZ'ADEK, M. D.,
KIEFF, RUSSIA.

The extraordinary difficulty of healing some cases of gleet is well known. There are men who have every morning an exudation, although they have tried every possible remedy against the evil. Frequently, especially after coitus or excess in saccho, the chronic disease becomes acute, which often leads to disagreeable results.

The product of the chronic inflammation of the urethra is an infiltration of the cells of the diseased mucous membrane ; the infiltration gradually becomes fibrous tissue, which, in its turn, produces the cicatrization called stricture ; the upper cells of this stratum, constantly sloughing, exfoliating and mingling with the mucous secretion of the glands, constitute the exudation which characterizes chronic gonorrhœa (gleet) ; the glands and lacunæ of the urethra are also affected. It is clear that many chronic gonorrhœas already represent the first stages of stricture, or, as Otis calls them "large sized strictures," which naturally cannot be cured by astringent solutions, or even such as are fatal to gonococci. If they are to be cured local pressure must be used to remove the cell infiltration, while medical treatment is adopted to affect the catarrh. For these reasons treatment with metallic bougies against chronic gonorrhœa has been mostly practiced.

In four years (1887–1890) I have treated fifty (50) cases of inveterate gleet with most satisfactory results, by the introduction into the urethra of Beniqué's duly curved tin bougies, anointed with either Unna's

Ol. cacao.................... 1,0
Ceræ flavæ............... 2,0–5,0
Bals. peruv.................... 2,0
Arg. nit.......................... 1,0
M.; f. ung.

Or Sperling's Salve:

Lanolini......................... 20,0

Ceræ albæ...................... 4,0

Arg. nit.................. 0,1–0,3

M.; f. ung.

These bougies are slightly conical and eleven centimeters in length. In nervous or very sensitive subjects, in whom the insertion of the bougies gives rise to pain and erection, from 3 to 5 per cent. solution of cocaine should be previously injected into the urethra ; during the first sitting the bougie is left *in situ* not longer than for three or five minutes, but later on the duration of the sitting is gradually increased up to fifteen or even twenty minutes. The sittings are repeated every two or three days, except in very inveterate and atonic cases, in which a daily introduction of the instrument is advisable. I have used a set of the bougies from No. 18 to No. 30 charriere, commencing with the least irritating bougies and gradually ascending to even larger ones. In relatively recent and in old cases I have usually employed Nos. 22, 24 and 25, while in more severe and protracted ones (which, by the way, constitute a vast majority) I have passed, following Professor Otis's instance, by degrees to No. 28 or even No. 30. In patients with narrowed ureter, I have enlarged the latter by means of a bistoury. In such cases where the lesions are situated in the bulbous or the posterior division of the urethra, his bougies should be introduced even as far backwards as the bladder ; according to my experience, the deep insertion, when practiced with due caution and carefulness, never causes any untoward accessory effects (such as vesical or urethral irritation, urethral fever, etc.) The therapeutic results which I have obtained with this treatment are satisfactory. My fifty cases of gleet, which were treated after the mechanical method, in five cases of urethritis posterior, complicated with obstinate vesical affection, the results were far from being satisfactory ; but in the remaining forty-five the treatment by metallic bougies proved highly suceessful. In thirty of the fifty cases a complete cure ensued; in another case a considerable improvement was obtained, and the only symptom left was a scanty mucoid discharge recurring from time to time. In relatively recent or in old cases from four to eight sittings proved to be sufficient for effecting cure. In a majority of cases, however, two or three months' course was necessary for the purpose, while in some unusually severe case, complicated with multiple strictures where a relapse took place, another course was to be repeated some months later.

The same method in the shape of the insertion of Nos. 20 to 30 gave to me most gratifying results, also, in fifteen cases of sexual asthenia, with spermatorrhœa and incomplete sexual impotence.

I arrive at the following general conclusions :

1. The method under consideration proves very useful: in many cases of neurasthenic irritation of the urethral mucous membrane, and in many cases of inveterate gleet, especially in those characterized by infiltration and other morbid changes in the mucous membrane and submucous tissues.

2. It proves beneficial further in inveterate gleet of an atonic character, or in that kept up by local nervous disturbances.

3. In some cases, however, the mechanical treatment must be supplemented by a subsequent course of local astringent remedies.

4. The method is free from any untoward accessory effects; provided, it is practiced with due precautions.

